# Prevalence and Clinical Characteristics of Patients with Pause-Dependent Atrioventricular Block

**DOI:** 10.3390/jcm11020449

**Published:** 2022-01-16

**Authors:** Sok-Sithikun Bun, Florian Asarisi, Nathan Heme, Fabien Squara, Didier Scarlatti, Philippe Taghji, Jean-Claude Deharo, Pamela Moceri, Emile Ferrari

**Affiliations:** 1 Cardiology Department, Pasteur University Hospital, Côte-d’Azur University, 06000 Nice, France; asarisi.f@chu-nice.fr (F.A.); heme.n@chu-nice.fr (N.H.); squara.f@chu-nice.fr (F.S.); scarlatti.d@chu-nice.fr (D.S.); moceri.p@chu-nice.fr (P.M.); ferrari.e@chu-nice.fr (E.F.); 2 Cardiology Department, Timone University Hospital, 13385 Marseille, France; philippetaghji@hotmail.com (P.T.); jean-claude.deharo@ap-hm.fr (J.-C.D.)

**Keywords:** pause-dependent atrioventricular block, high-grade atrioventricular block, prevalence, syncope, pacemaker

## Abstract

Background: In patients with complete atrioventricular block (AVB), the prevalence and clinical characteristics of patients with pause-dependent AVB (PD-AVB) is not known. Our objective was to assess the prevalence of PD-AVB in a population of patients with complete (or high-grade) AVB. Methods: Twelve-lead electrocardiogram (ECG) and/or telemonitoring from patients admitted (from September 2020 to November 2021) for complete (or high-degree) AVB were prospectively collected at the University Hospital of Nice. The ECG tracings were analyzed by an electrophysiologist to determine the underlying mechanism of PD-AVB. Results: 100 patients were admitted for complete (or high-grade) AVB (men 55%; 82 ± 12 years). Arterial hypertension was present in 68% of the patients. Baseline QRS width was 117 ± 32 ms, and mean left ventricular ejection fraction was 56 ± 7%. Fourteen patients (14%) with PD-AVB were identified, and presented similar clinical characteristics in comparison with patients without PD-AVB, except for syncope (which was present in 86% versus 51% in the non-PD-AVB patients, *p* = 0.01). PD-AVB sequence was induced by: Premature atrial contraction (8/14), premature ventricular contraction (5/14), His extrasystole (1/14), conduction block in a branch (1/14), and atrial tachycardia termination (1/14). All patients with PD-AVB received a dual-chamber pacemaker during hospitalization. Conclusion: The prevalence of PD-AVB was 14%, and may be underestimated. PD-AVB episodes were more likely associated with syncope in comparison with patients without PD-AVB.

## 1. Introduction

Pause-dependent atrioventricular block (PD-AVB) can be defined as a paroxysmal and complete AVB whose mechanism is “bradycardia-dependent”. An early description of bradycardia-dependent AVB was made in 1971 by Coumel et al. [1], and a few years later, the mechanism of PD-AVB was reported by Rosenbaum et al. [2]. Although some recent publications use the term phase 4 AVB, a controversy still exists about the exact mechanism of PD-AVB. [3] Actually, a recent report suggested the possible functional nature of PD-AVB related to concealed retrograde conduction in non-diseased His–Purkinje structures [4]. Nevertheless, despite the existence of variable triggers, “pause-dependency” remains the common hallmark. PD-AVB occurrence is related to the inactivation of sodium channels due to spontaneous depolarization within diseased His–Purkinje fibers, during a post-compensatory pause, followed by inability to conduct electrical impulses due to a very low resting membrane potential (phase 4 of action potential). This mechanism may be distinguished from other types of complete AVB. In 2009, Lee et al. proposed a classification of complete AVB in four types: Acquired AVB, vagally-mediated AVB, congenital heart block, and paroxysmal AVB, the later referring to PD-AVB [5]. Recently, the different triggers of PD-AVB have been well described: Premature atrial contraction (PAC), premature ventricular contraction (PVC), supra-ventricular tachycardia termination, sinus rhythm slowdown [6].

PD-AVB is considered a rare entity, but the prevalence of 4AVB is not known. The purpose of this study was to assess the incidence and the clinical characteristics of patients with PD-AVB in a population of patients admitted into a cardiac care unit for complete (or high-grade) AVB.

## 2. Materials and Methods

**Figure 1:**
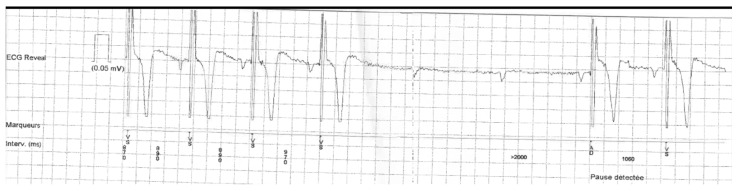
Example of patient presenting with vagally-mediated complete AVB recorded with an implantable loop recorder. One can notice the gradual PP lengthening before the occurrence of complete AVB, but also during the absence of the QRS complexes. A PR interval prolongation is also visible before AVB occurred, with the PR interval after resolution of the block slightly longer than PR before the block. This type of AVB mechanism was excluded from the study. AVB: atrioventricular block.

All patients admitted at the cardiac care unit of the University Hospital of Nice from September 2020 to November 2021 for complete (or high-grade) AVB were included in this single-center prospective observational study, and their electrocardiograms (ECGs) were systematically collected. Complete AVB but also high-grade (including 2 to 1) AVB were confirmed on 12-lead surface and/or telemetry monitoring by one experienced electrophysiologist (S.-S.B.). Patients with vagally-mediated AVB (Figure 1) defined by gradual slowing of the sinus rate (PP interval lengthening) and AV conduction (prolonging PR) usually seen before complete AVB occurrence, were excluded from this analysis.

AVB were classified as permanent if complete (or high-grade) AVB was present all along the period of the hospitalization stay during cardiac monitoring, or intermittent if complete (or high-grade) AVB was observed in alternation with 1:1 conduction (eventually facilitated by isoproterenol infusion). To avoid any confusion with previous studies, the term “paroxysmal” will not be used in our study and will be discussed later.

The clinical and electrocardiographic characteristics of the patients were analyzed; episodes of PD-AVB were classified according to their initiation mechanism. All patients gave their written informed consent for the pacemaker implantation (if needed). The study was approved by the institutional review board.

### Statistical Analysis

The statistical analysis was made with Excel (San Diego, CA, USA). Categorical variables are described as numbers and percentages. Continuous variables are described as mean ± SD for variables with normal distributions or as median with range for variables not normally distributed.

## 3. Results

**Figure 2:**
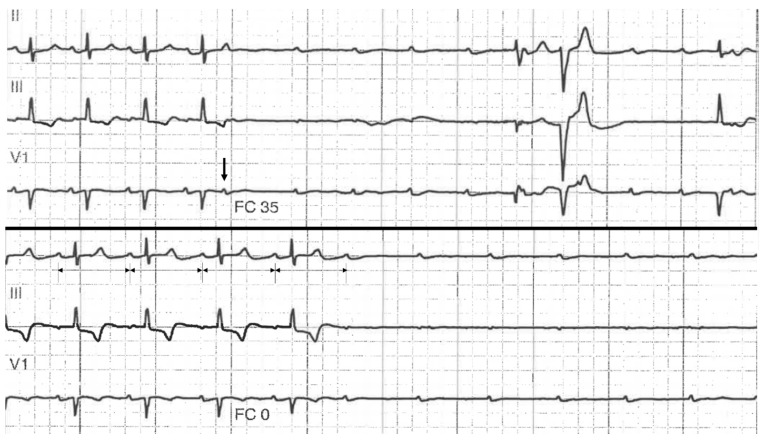
Example of patient presenting with PD-AVB initiated by a premature atrial contraction, recorded on a telemetry strip (upper panel). Another episode could be recorded in the same patient (patient 5 from Table 2), but without any macroscopic variation of the PP intervals preceding the complete AVB (lower panel).

**Figure 3:**
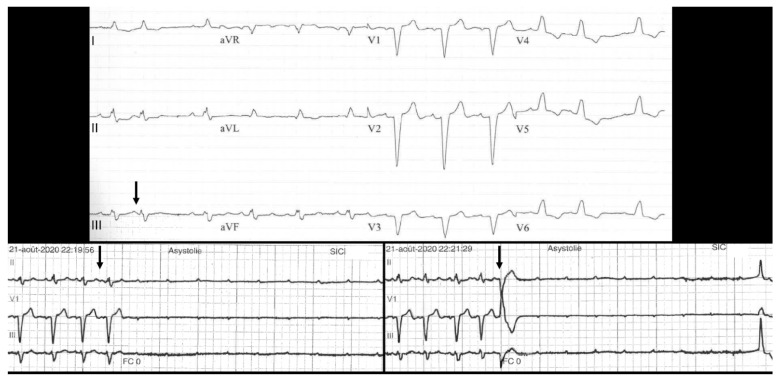
Example of two mechanisms of pause-dependent atrioventricular block seen within the same patient (patient 10 in Table 2). Baseline twelve-lead ECG shows sinus rhythm with complete left bundle branch block and a conducted premature atrial contraction (PAC). Another timely-appropriate PAC then induced PD-AVB (left lower image). Another PD-AVB episode was recorded after a premature ventricular contraction (lower right image).

**Figure 4:**
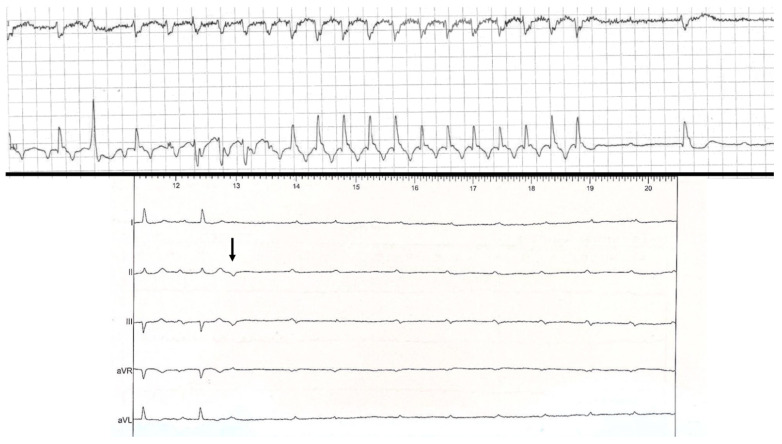
Example of two other mechanisms of pause-dependent atrioventricular blocks. The telemetry strips show a counterclockwise isthmus-dependent flutter termination followed by PD-AVB (upper panel). In the same patient (11 from Table 2), another episode could be recorded, induced by a premature ventricular contraction. Surface ECG showed a PD-AVB episode induced by a His extrasystole (arrow) as shown by the retrograde P wave visible (lower panel). A blocked premature atrial contraction may not be ruled out (patient 13 in Table 2).

During the inclusion period, 100 patients were admitted for complete (or high-grade) AVB (men 55%; 82 ± 12 years). Arterial hypertension was present in 68% of the patients. Baseline QRS width was 117 ± 32 ms, and mean left ventricular ejection fraction was 56 ± 7%. Fourteen patients (14%) with PD-AVB were identified, and presented similar clinical characteristics in comparison with patients without PD-AVB except for syncope (present in 86% versus 51% in the non-4AVB patients) (Table 1). PD-AVB sequence was induced by: Premature atrial contraction (8/14), as shown in Figure 2; premature ventricular contraction (5/14), as seen in Figure 3; His extrasystole (1/14); conduction block in a branch (1/14); and atrial tachycardia termination (1/14), as shown in Figure 4. The mean duration of asystoly during the PD-AVB episode was 5.1 ± 3 s (1.6–10 s). The mean number of consecutive non-conducted p waves during the PD-AVB episode was 8 ± 4 (range 3–15). Patients with PD-AVB represented one third of the total number (*n* = 43) of patients with intermittent complete AVB. Baseline conduction disturbances in patients with intermittent complete AVB are presented in Table 1. All of the patients with PD-AVB received a dual-chamber pacemaker during hospitalization. After a mean follow-up of 6 ± 7 months, the mean percentage of ventricular pacing was 89.2 ± 30% in this population (complete AVB present except for two, one with initial baseline normal QRS, and the other with bifascicular block); versus 73.4 ± 39% in the non-PD-AVB group (*p* = 0.31).

## 4. Discussion

Our study is the first to report the prevalence of PD-AVB in a population of patients admitted into a cardiac care unit for complete (or high-grade) AVB. While 4AVB was considered a rare phenomenon, our study demonstrates a prevalence of 14%, reaching one third if considering only patients with intermittent complete (or high-grade) AVB. Our cohort of patients is representative of the prevalence of complete (or high-grade) AVB in a University center with medium-to high-volume activity. Isolated descriptions or case reports/case series have been published concerning PD-AVB, without further information about its prevalence [7]. In our study population, no specific clinical or electrocardiographic characteristics could be related to the occurrence of PD-AVB, in comparison with other patients with complete AVB. Syncope was significantly more frequently present in patients with PD-AVB, in comparison with patients without PD-AVB (86 versus 51%, *p* = 0.01). To the best of our knowledge, our series is the second largest described with PD-AVB to date. Of note, a recent case reported the possibility for conduction block to occur within a bundle branch, and not within the AV node itself [12]. All previously published cases are summarized in Table 2 [8].

**Figure 5:**
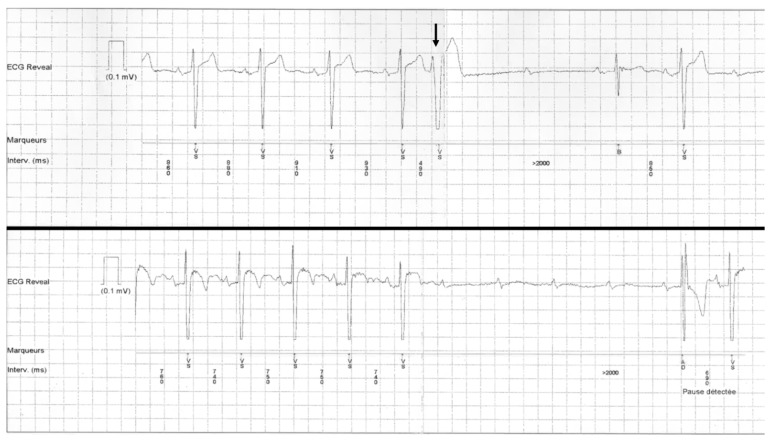
Example of pause-dependent atrioventricular block induced by a premature ventricular contraction (arrow), and recorded with an implantable loop recorder. In the same patient, another episode was preceded by sinus rhythm acceleration (from 760 to 740 ms) corresponding to tachycardia-dependent atrioventricular block.

Interestingly, our study is the first to report double distinct mechanisms recorded within the same patient. Four patients presented with two distinct mechanisms of PD-AVB: One patient had PVC-induced PD-AVB and a second episode without significant increase in the PP intervals before AVB (from 690 to 710 ms), and without PR prolongation (Figure 2); another patient presented PD-AVB sequences induced by PAC, and later by PVC (Figure 3); the third patient presented an episode triggered by atrial flutter termination and PVC-induced PD-AVB; the last patient presented PVC-induced PD-AVB and another recorded episode with sinus rhythm acceleration (tachycardia-dependent AVB) (Figure 5). Uhm et al. reported a dual mechanism in a patient presenting PD-AVB episodes induced by a PAC, and SVT termination, but the later episode included the intervention of a temporary pacemaker (not spontaneous) [6].

Lee et al. reported the largest bicentric series to date of paroxysmal AVB. Nevertheless, both tachycardia-dependent AVB [5] and idiopathic paroxysmal AVB were not clearly individualized in this initial description. Actually, a few years later, Brignole et al. described a rare entity, and reported a series of 18 patients with idiopathic paroxysmal AVB, whose mechanism is related to adenosine hypersensitivity [13,14].

**Figure 6:**
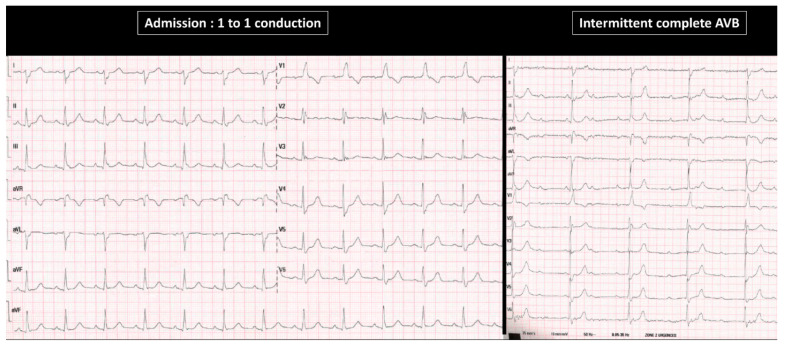
Twelve-lead ECG from an 82 year-old female patient admitted at the emergency department for syncope with cerebral traumatism. The initial ECG shows sinus rhythm with 1 to 1 atrioventricular conduction and complete right bundle branch block associated with left fascicular posterior block. During ECG monitoring at the cardiac care unit, the patient developed an intermittent complete “intrinsic” AVB episode. No trigger of PD-AVB was found in this patient, who was included in the non-PD-AVB group.

To summarize, our suggestion for paroxysmal AVB classification may be as follows:

(1) “Extrinsic” vagally-mediated AVB characterized by significant PR prolongation or Wenckebach before initiation of AVB, gradual slowing of the sinus rate (PP interval), resumption of AV conduction with sinus acceleration, PP interval prolongation during ventricular asystole. Often, a shortening of the PR interval compared to the last PR interval with AV conduction before an AV block can be observed (upon withdrawal of the vagal effect). A clinical history suggestive of heightened vagal tone is present.
(2) “Idiopathic” paroxysmal AVB involving a younger population (mean age 55 ± 19 years) without cardiac and ECG abnormalities, without progression to persistent forms of AVB, and with efficacy of cardiac pacing. AVB occurs with abrupt onset and delayed emergence of an adequate escape rhythm without PP cycle lenghthening or PR interval prolongation. A low baseline adenosine plasma level was found in this specific population. This entity should remain a diagnosis of exclusion.
(3) “Intrinsic” AVB (suggesting AV conduction disease), which may be divided into four categories: Congenital heart block; tachycardia-dependent AVB; PD-AVB; and finally other acquired AVB when the preceding features/conditions are lacking (non-PD-AVB group in our study) as shown in Figure 6. Progressive cardiac conduction disease may be integrated into this last category, and refers to primary genetic degenerative diseases of genetic origin (several mutations have been described, such as in SCN5A of the cardiac sodium channel) [9]. Combined AVB initiation circumstances may be encountered in this “intrinsic” AVB group.

The main differences that may be seen between our population and Lee’s report are that their population was significantly younger than ours (69 versus 84 ± 6 years-old) with thinner QRS duration (123 ± 32 versus 132 ± 27 ms in our series). The authors also reported that PAC was the main trigger of PD-AVB in 30% of the cases, PVC in 23%, His extrasystole in 10%, and other variable mechanisms in 37%. Our results are in line with the literature, because PAC were also found to be the most prominent trigger (two thirds).

Of note, in our series, two patients out of 14 with PD-AVB underwent a transcatheter aortic valve intervention (TAVI) in their past medical history. According to the latest European guidelines, early permanent cardiac pacing may be recommended in the case of transient high-grade AVB during TAVI in patients with pre-existing RBBB [15,16]. Actually, transitory AVB may usually be encountered during rapid ventricular pacing before valve expansion. In this case, permanent pacemaker implantation could then be discussed if baseline RBBB was present before the intervention. For patients with newly-developed LBBB, an ambulatory ECG monitoring or electrophysiological study may be considered.

### Limitations

This is a monocentric study with a limited number of patients. True prevalence of PD-AVB could have been underestimated. No electrophysiological study was performed in this elderly population with an indication of permanent cardiac pacing (syncope present in 80% of the cases). Electrophysiological study is usually not recommended in the setting of symptomatic complete AVB [16].

Of note, all our patients with PD-AVB received a dual-chamber pacemaker (with exclusive right ventricular septal lead position). PD-AVB (once identified) usually occurs in diseased His–Purkinje systems. Further studies are needed to confirm the usefulness/superiority of conduction system pacing in these patients, as recently reported by Du et al. [4].

## 5. Conclusions

The prevalence of 4AVB was 14%, and may be underestimated. PD-AVB episodes were more likely associated with syncope in comparison with patients without PD-AVB. These patients may elicit multiple triggers of PD-AVB.

## Figures and Tables

**Table 1:** Characteristics of patients with pause-dependent atrioventricular block.

	PD-AVB ^1^ (*n* = 14)	Non-PD-AVB (*n* = 86)	*p*
Age	84 ± 6 (71–96)	82 ± 12 (57–102)	0.22
Men, *n* (%)	9 (64)	46 (53)	0.45
Syncope	12 (86)	44 (51)	0.01
Arterial hypertension, *n* (%)	10 (71)	58 (67)	0.77
LVEF (%) ²	55 ± 10	56 ± 7	0.57
Mean QRS duration (ms)	132 ± 27	117 ± 32	0.15
PR interval (ms)	230 ± 36	199 ± 43	0.10
RBBB ^3^/LBBB ^4^/Normal QRS, *n* (%)	RBBB = 3 (22) RBBB/LAFB ^5^ = 3 (22) RBBB/LPFB ^6^ = 2 (14) LBBB = 2 (14) Normal = 4 (28)	RBBB = 2 (7) RBBB/LAFB = 4 (14) RBBB/LPFB = 5 (17) LBBB = 5 (17) Normal = 12 (41) Isolated LAFB = 1 (4)	0.16 0.34 0.80 0.80 0.42
Baseline corrected QT interval (ms)	453 ± 44	470 ± 53	0.64
Tpeak-Tend (ms)	114 ± 48	117 ± 45	0.69

^1^ PD-AVB: pause-dependent atrioventricular block. ² LVEF: left ventricular ejection fraction. ^3^ RBBB: right bundle branch block. ^4^ LBBB: left bundle branch block. ^5^ LAFB: left anterior fascicular block. ^6^ LPFB: left posterior fascicular block.

**Table 2:** Summary of published cases of pause-dependent atrioventricular block.

Author	Sex	Age (y)	Baseline QRS Width (ms)	Symptom	Mechanism	Outcome
Lee S, 2009 [5]	NA *n* = 30	69	123 ± 32	Syncope (75%)	PAC ^1^ (30%) PVC ^2^ (23%) His extrasystole (10%) Other (37%)	Pacemaker
Atreya AR, 2015 [7]	Male	NA ^3^	96	Syncope	PAC	Pacemaker
Georger F, 2015 [8]	Male	74	NA	Syncope	AT termination	Pacemaker
Shesana M, 2017 [9]	Male	45	130	Syncope	PAC	Pacemaker
Bansal R, 2017 [10]	Female	79	Narrow	Near-syncope	PVC	Pacemaker
Prasada S, 2019 [11]	Male	81	130	None	PVC	Pacemaker
Uhm JS, 2018 [6]	Male	72	Narrow	Syncope	Vagally-mediated	Pacemaker
	Male	73	NA	Asymptomatic	PVC	Pacemaker refused
	Male	69	Narrow	Syncope	PVC	No pacemaker
	Male	68	NA	Dizziness	PAC	Pacemaker
	Male	71	Narrow	Syncope	AT termination	Pacemaker
Du W, 2020 [3]	Male	76	130	Dizziness	PVC	Pacemaker
Our series, 2022	Female	96	134	Asymptomatic	Block in branch	Pacemaker
	Male	88	156	Syncope	PAC	Pacemaker
	Female	87	112	Syncope	PAC	Pacemaker
	Female	91	124	Heart failure	PVC	Pacemaker
	Female	91	74	Syncope	PVC	Pacemaker
	Female	79	122	Syncope	PAC	Pacemaker
	Male	84	156	Syncope	PAC	Pacemaker
	Male	90	170	Syncope	PAC	Pacemaker
	Male	83	160	Syncope	PVC	Pacemaker
	Male	86	144	Syncope	PAC/PVC	Pacemaker
	Male	76	140	Syncope	AFl ^4^ termination/PVC	Pacemaker
	Male	72	115	Syncope	PVC/SR acceleration ^5^	Pacemaker
	Male	81	96	Syncope	His extrasystole	Pacemaker
	Male	71	152	Syncope	PAC	Pacemaker

^1^ PAC: premature atrial contraction. ² PVC: premature ventricular contraction. ^3^ NA: non-available. ^4^ AFl: atrial flutter. ^5^ SR: sinus rhythm.
